# Transarterial chemoembolization combined with molecular targeted agents plus immune checkpoint inhibitors for unresectable hepatocellular carcinoma beyond the up-to-seven criteria: a propensity score-matching analysis

**DOI:** 10.1080/07853890.2024.2419993

**Published:** 2024-11-01

**Authors:** Wen Chen, Hai-Tao Yan, Jin-Xing Zhang, Chun-Gao Zhou, Jin Liu, Sheng Liu, Hai-Bin Shi, Yuan Cheng, Qing-Quan Zu

**Affiliations:** a Department of Interventional Radiology, The First Affiliated Hospital with Nanjing Medical University, Nanjing, China; b Department of Clinical Medicine Research Institution, The First Affiliated Hospital with Nanjing Medical University, Nanjing, China; c Department of Medical Oncology, Jinling Hospital, Affiliated Hospital of Medical School, Nanjing University, Nanjing, China

**Keywords:** Carcinoma, hepatocellular, chemoembolization, therapeutic, molecular-targeted therapy, immune checkpoint inhibitors

## Abstract

**Purpose:**

Not all patients benefit from transarterial chemoembolization (TACE) due to the heterogeneity of the tumour burden in intermediate-stage hepatocellular carcinoma (HCC). To compare the outcomes of transarterial chemoembolization (TACE) combined with molecular-targeted agents plus immune checkpoint inhibitors (TACE-MTAs-ICIs) with those of TACE for patients with unresectable hepatocellular carcinoma (uHCC) that were beyond the up-to-seven criteria.

**Patients and methods:**

Between January 2019 and July 2022, 130 patients diagnosed with uHCC beyond the up-to-seven criteria were retrospectively identified, including 47 patients who received TACE-MTAs-ICIs and 83 patients who received TACE alone. The primary endpoints were overall survival (OS) and progression-free survival (PFS); the secondary endpoints included tumour response and adverse events (AEs).

**Results:**

There were 43 matched patients. The median OS and PFS times in the TACE-MTAs-ICIs group were significantly longer than those in the TACE group (OS: 27.2 vs. 15.9 months, *p* = 0.007; PFS: 15.4 months vs. 4.8 months, *p* < 0.001). The objective response rate (ORR) in the TACE-MTAs-ICIs group was higher than that in the TACE group (65.1% vs. 37.2%, *p* = 0.010). Reversible AEs (grade 3 or 4) occurred differently in TACE-MTAs-ICIs and TACE groups (83.7% vs. 51.2%, *p* = 0.001). Univariate and multivariate analyses revealed that TACE-MTAs-ICIs treatment was an independent favourable prognostic factor for both PFS and OS (*p* < 0.001).

**Conclusion:**

For uHCC patients beyond the up-to-seven criteria, TACE-MTAs-ICIs provided superior ORR and OS. Early combined TACE and systemic treatment should shift for patients who are beyond these criteria.

## KEY MESSAGES

1. The ORR and median OS reached 65.1% and 27.2 months, respectively, in the treatment model of TACE-MTAs-ICIs for patients with unresectable HCC that were beyond the up-to-seven criteria.
2. TACE-TKI-ICI yielded a synergistic effect on these patients and was an independent favourable prognostic factor for PFS and OS.

## Introduction

Hepatocellular carcinoma (HCC) is the sixth most common malignancy worldwide and represents a major global healthcare challenge [1]. Most HCC patients are initially diagnosed at an intermediate or advanced stage, which means that they miss the opportunity for curative treatment [2]. Transarterial chemoembolization (TACE) has been recommended as the primary treatment for patients with intermediate-stage HCC [3, 4]. Due to the heterogeneity of tumour burden and liver function in intermediate-stage HCC, not all patients experience the same benefits from TACE [5].

The up-to-seven criteria (the sum of the size (cm) of the largest tumour and the number of tumours) are commonly used to define Barcelona Clinic Liver Cancer (BCLC) B-stage HCC [6–8], and the efficacy of TACE alone is unsatisfactory for HCC patients meeting the beyond the up-to-seven criteria [9, 10]. The objective response rate (ORR) was 37.8% in patients treated with TACE alone [10]. TACE refractoriness also occurred earlier in patients beyond the up-to-seven criteria than in patients within the criteria (the median time to untreatable progression was 25.7 vs. 14.6 months, *p* = 0.005) [11]. A proof-of-concept study reported that switching patients who were beyond the up-to-seven criteria to lenvatinib contributed to improved prognosis [median overall survival (OS): 37.9 vs. 21.3 months, *p* < 0.01] [9]. In addition, the TACE procedure could exacerbate microenvironmental hypoxia and stimulate vascular endothelial growth factor (VEGF) and fibroblast growth factor [12–14], which compromised the clinical benefit. Therefore, a previous study explored whether TACE combined with molecular-targeted agents (MTAs) could improve the clinical outcomes of intermediate-stage HCC patients. In the specific subgroup of patients who were beyond the up-to-seven criteria, no significant improvement was found. As for systemic therapies, one prior study showed that the ORR at 6 weeks of atezolizumab plus bevacizumab in patients with HCC beyond the up-to-seven criteria was 42.5% according to the modified response evaluation criteria in solid tumours (mRECIST) [15]. Therefore, for intermediate-stage patients beyond the up-to-seven criteria, treatment remains challenging. Notably, immune checkpoint inhibitors (ICIs) have exhibited promising clinical benefits and play a vital role in the long-term remission of tumours [16, 17]. Considering the secondary enhanced PD-1/PD-L1 expression after TACE [18], in theory, combining ICIs with TACE and MTAs can reduce neovascularization and prolong immune activation in patients with HCC, which results in a synergistic effect on the antitumor response [16, 17]. Based on these findings, we hypothesized that combined TACE with MTAs and ICIs would provide a superior clinical benefit for patients with HCC beyond the up-to-seven criteria.

Therefore, we conducted this retrospective study to evaluate the efficacy and safety of combining TACE with MTAs plus ICIs for HCC patients beyond the up-to-seven criteria.

## Patients and methods

This retrospective study was approved by the local institutional ethics review board. Written informed consent was not required for this retrospective study. The study was conducted by the World Medical Association Declaration of Helsinki and was approved by the Ethics Committee of our institution. The authors confirm that the data supporting the findings of this study are available within the article and its supplementary materials.

### Patients

**Figure 1.**
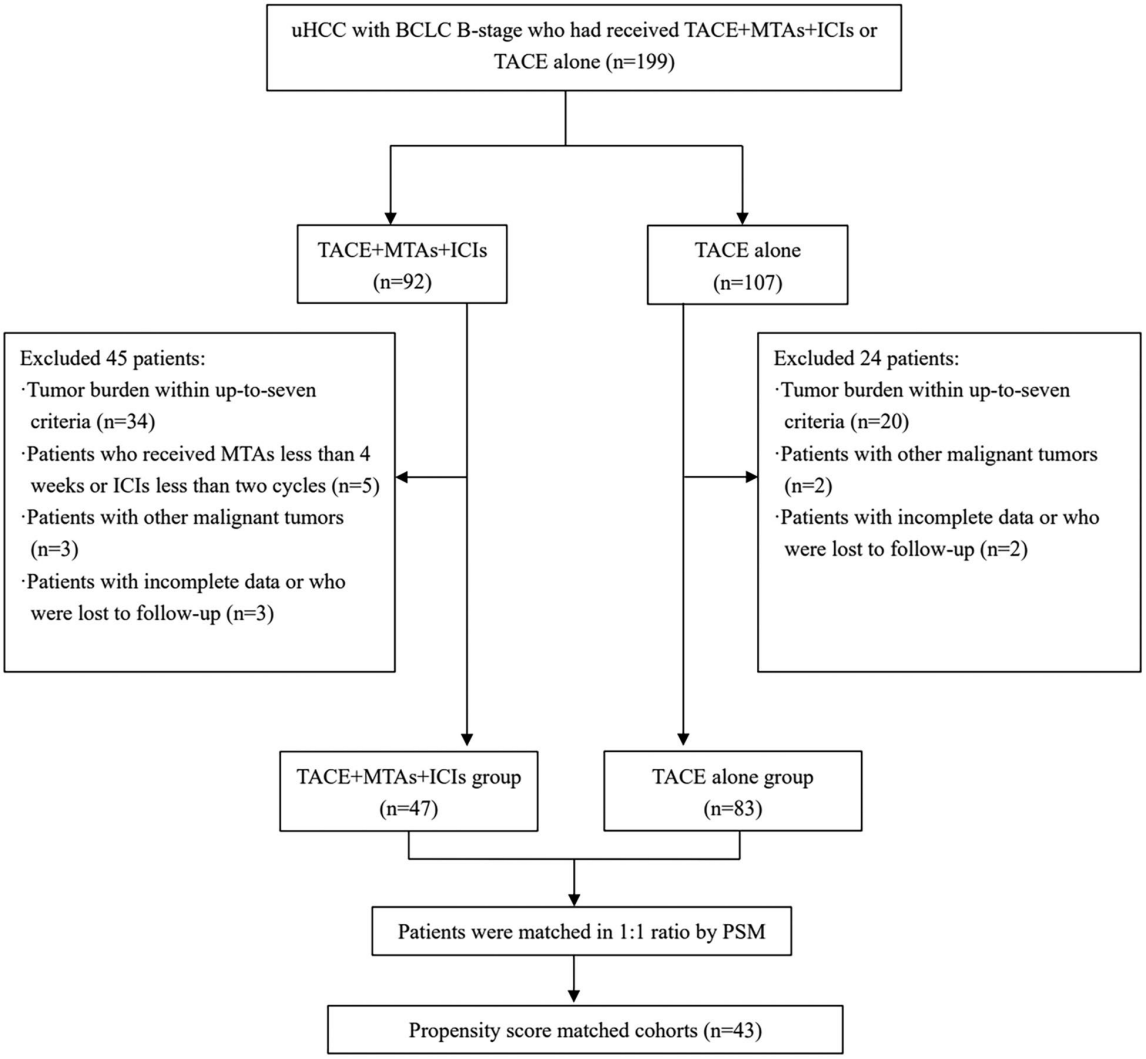
Patient enrolment flowchart. Abbreviations: uHCC, unresectable hepatocellular carcinoma; BCLC, Barcelona Clinic Liver Cancer; TACE, transarterial chemoembolization; MTAs, molecular targeted agents; ICIs, immune check-point inhibitors; PSM, propensity score matching.

Between January 2019 and June 2022, the medical records of 199 patients were reviewed. HCC was confirmed pathologically or clinically based on the Guidelines for Diagnosis and Treatment of Primary Liver Cancer in China [3]. The inclusion criteria were as follows: (1) BCLC B-stage patients; (2) Eastern Cooperative Oncology Group (ECOG) performance status score of 0; and (3) Child-Pugh class A or B. The exclusion criteria were as follows: (1) tumour burden within the up-to-seven criteria; (2) patients who received MTAs less than 4 weeks or ICIs less than two cycles; (3) patients with other malignant tumours; and (4) patients with incomplete data or who were lost to follow-up. In this study, there were overlapping population with our previous research (21 patients who treated with TACE alone) [14]. A total of 130 patients were ultimately included in this study (Figure 1).

Base class, albumin-bilirubin (ALBI) grade, α-fetoprotein (AFP) level, ECOG performance status, number of tumours, maximum tumour diameter, and subclassification of BCLC B-stage [7].

### TACE procedure

TACE was performed before systemic therapy. The TACE procedure was performed in a superselective fashion. A 5-F catheter was introduced under local anaesthesia, and then, angiography was used to identify the tumour number, size, and location. Then, a chemotherapeutic agent (lobaplatin, 30 to 50 mg) was infused through the hepatic artery. Subsequently, a super-selective microcatheter was inserted into the feeding artery of the tumours. The embolization procedure using a microcatheter was conducted employing a standard technique involving lipiodol (5 to 20 mL) combined with epirubicin (10 to 20 mg). The feeding arteries were blocked by gelatin sponges or particles. The TACE procedure was performed by two doctors with over 10 years of experience.

### Systematic therapy

MTAs or ICIs were suspended during the TACE procedure, and patients recovered after TACE. Atezolizumab (1200 mg), sintilimab (200 mg), or camrelizumab (200 mg) were injected intravenously approximately every 3 weeks. For MTAs, bevacizumab was injected intravenously at 7.5 mg/kg, and lenvatinib was administered orally at a dose of 8 mg for patients < 60 kg or 12 mg for patients ≥ 60 kg daily. Termination or changes to the therapeutic regimen were considered based on disease progression, unacceptable adverse events (AEs), patient refusal, or clinician decision.

### Follow-up and assessments

Overall survival (OS) was defined from the date of the initiation of therapy to the date of death for any reason; progression-free survival (PFS) was defined from the date of the initiation of therapy to the date of progression. Enhanced CT or MRI was implemented at 2–3-month intervals. Iodixanol is used for dynamic three-phase scans in enhanced CT, with scans conducted at 25 ∼ 30 s, 60 ∼ 75 s, and 120 ∼ 180 s for the arterial, portal venous, and delayed phases, respectively. Gadolinium ethoxybenzyl diethylenetriaminepentaacetic acid is used for dynamic four-phase enhanced MRI scans, and post-contrast images are obtained at 15 s, 45 s, 3 min, and 15 min after contrast injection for arterial, portal venous, delayed, and hepatobiliary phases. All image evaluations were performed by a radiologist with over 8 years of experience. Tumour responses were assessed based on the mRECIST [19]. The objective response rate (ORR) was defined as the proportion of patients who achieved a complete response (CR) or partial response (PR). The disease control rate (DCR) was defined as the rate of objective response plus stable disease (SD).

Treatment-related toxicity was observed and recorded according to the National Cancer Institute Common Toxicity Criteria Adverse Events (CTCAE) version 5.0. Liver function, blood coagulation profile, and serum alpha-fetoprotein (AFP) levels were examined approximately 1 month later. The end date of the follow-up was 31 October 2022.

### Statistical analysis

A propensity score matching (PSM) analysis was conducted between the TACE-MTAs-ICIs group and the TACE group to minimize study bias, and the caliper value was set at 0.02. The variables were as follows: age, sex, Child-Pugh class, AFP, BCLC B substage, and aetiology. OS and PFS were estimated using the Kaplan–Meier method, and the differences were assessed for significance using the log-rank test. The Cox proportional hazards regression model was used to determine the factors associated with survival. Factors with *p* < 0.1 in the univariate analysis were input into the multivariate analysis. Categorical data are expressed as percentages and frequencies and were compared between the two groups using the χ2 test or Fisher’s exact test as appropriate. A two-tailed *p* value < 0.05 was considered statistically significant. Statistical analysis was performed using SPSS (Version 25.0, Chicago, IL) and GraphPad Software (Prism 8.0.1, San Diego, California).

## Results

### Patient characteristics

A total of 47 patients and 83 patients treated with TACE-MTAs-ICIs and TACE alone, respectively, were enrolled in this study. The clinical characteristics of all the patients (*n* = 130) are given in Table 1. After the PSM analysis, 43 pairs of patients were matched (Figure 1). The two groups were similar in each clinical variable after PSM. The average number of TACE procedures was 3.2 (range, 1–11) and 3.6 (range, 1–11) in the TACE-MTAs-ICIs and TACE groups, respectively.

**Table 1.** Patient characteristics before and after propensity score matching.

Variables	Before PSM			After PSM		
	TACE-MTAs-ICIs	TACE	*p* value	TACE-MTAs-ICIs	TACE	*p* value
	(*n* = 47)	(*n* = 83)		(*n* = 43)	(*n* = 43)	
Age (years)			0.222			0.664
≥ 60	18 (38.3%)	41 (49.4%)		18 (41.9%)	20 (46.5%)	
< 60	29 (61.7%)	42 (50.6%)		25 (58.1%)	23 (53.5%)	
Sex			0.102			1.000
Male	43 (91.5%)	67 (80.7%)		40 (93.0%)	39 (90.7%)	
Female	4 (8.5%)	16 (19.3%)		3 (7.0%)	4 (9.3%)	
Aetiology			0.041			0.178
HBV	39 (83.0%)	55 (66.3%)		36 (83.7%)	40 (93.0%)	
Others	8 (17.0%)	28 (33.7%)		7 (16.3%)	3 (7.0%)	
Child-Pugh Class			0.743			0.763
A	39 (83.0%)	69 (83.1%)		36 (83.7%)	37 (86.0%)	
B	8 (17.0%)	14 (16.9%)		7 (16.3%)	6 (14.0%)	
BCLC B substage			0.703			1.000
B2	44 (93.6%)	79 (95.2%)		42 (97.7%)	42 (97.7%)	
B3	3 (6.4%)	4 (4.8%)		1 (2.3%)	1 (2.3%)	
Tumour diameter (cm)			0.042			0.074
≥ 7	34 (72.9%)	45 (54.2%)		31 (72.1%)	23 (53.5%)	
< 7	13 (17.7%)	38 (45.8%)		12 (27.9%)	20 (46.5%)	
Tumours number			0.646			0.510
< 3	19 (40.4%)	37 (44.6%)		16 (37.2%)	19 (44.2%)	
≥ 3	28 (59.6%)	46 (55.4%)		27 (62.8%)	24 (55.8%)	
ALBI			0.330			0.357
1	12 (24.5%)	28 (33.7%)		12 (27.9%)	16 (37.2%)	
2	35 (74.5%)	55 (66.3%)		31 (72.1%)	27 (62.8%)	
AFP (ng/mL)			0.226			0.666
< 400	22 (46.8%)	48 (57.8%)		20 (46.5%)	22 (51.2%)	
≥ 400	25 (53.2%)	35(42.2%)		23 (53.5%)	21 (48.8%)	

Abbreviations: PSM, propensity score matching; HBV, hepatitis B virus; ALBI, albumin-bilirubin; AFP, α-fetoprotein; BCLC, Barcelona Clinic Liver Cancer.

### Survival analysis

**Figure 2.**
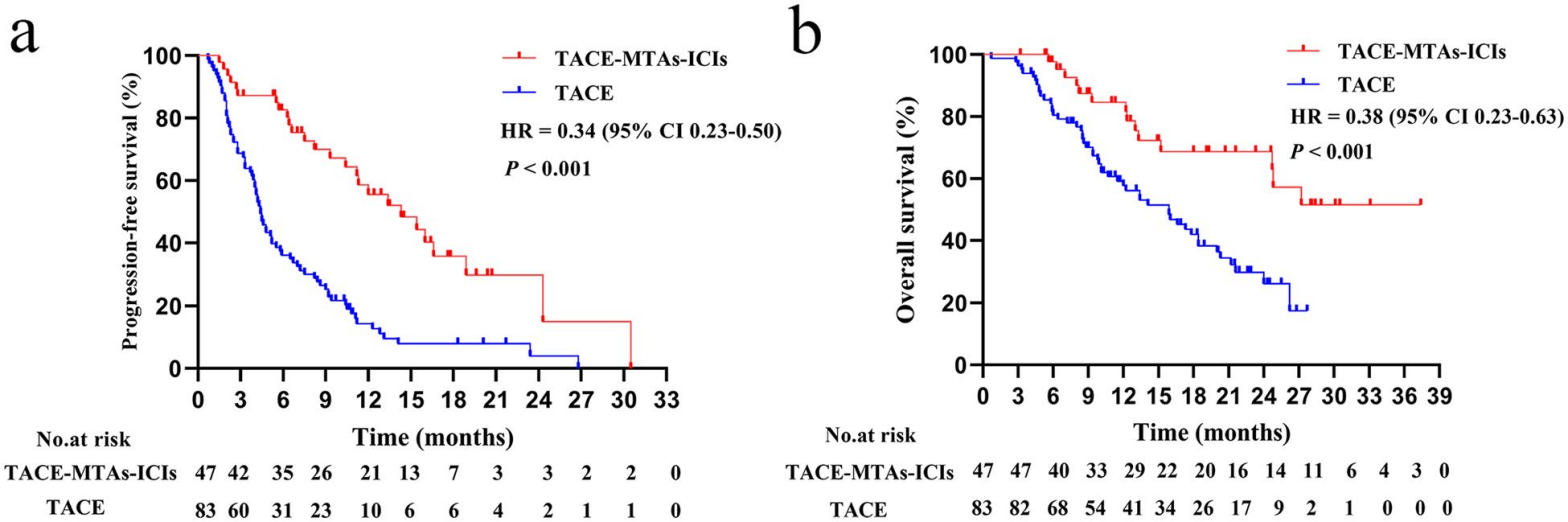
Kaplan–Meier survival for progression-free survival before PSM (a). Kaplan–Meier survival for survival before PSM (b).

**Figure 3.**
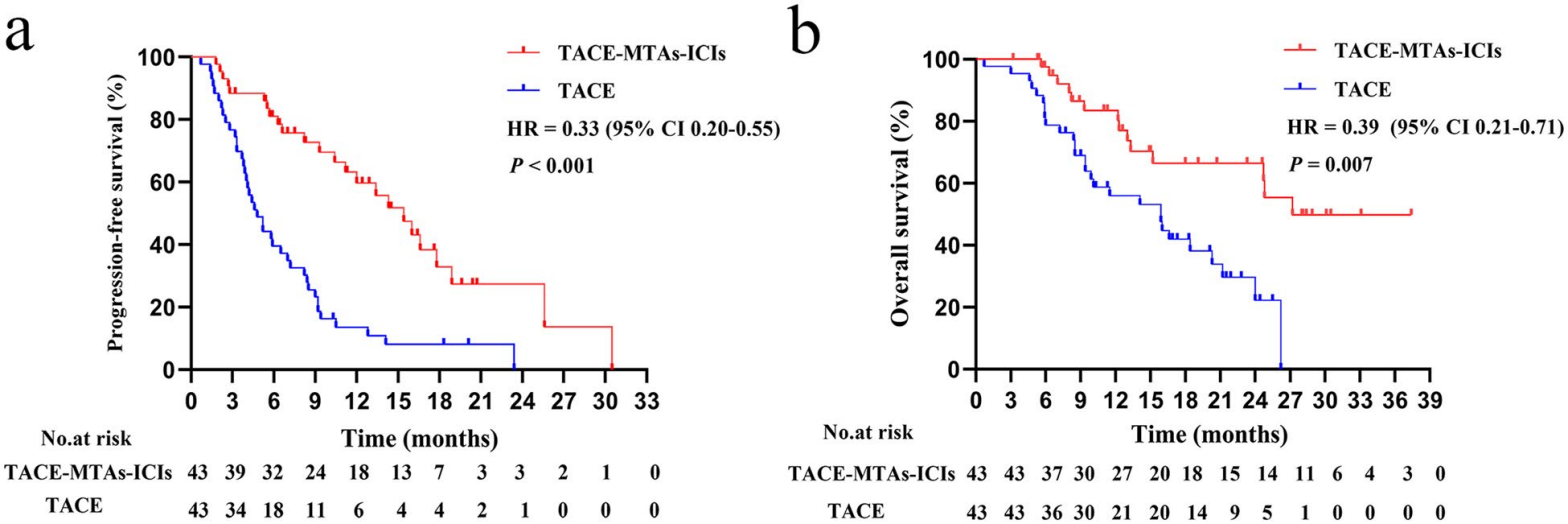
Kaplan–Meier survival for progression-free survival before PSM (a), Kaplan–Meier survival for overall survival after PSM (b).

The median follow-up was 21.6 months. Before PSM, 14 patients (29.8%) in the TACE-MTAs-ICIs group and 51 patients (61.4%) in the TACE group died. The median PFS was 14.3 months in the TACE-MTAs-ICIs group and 4.4 months in the TACE group (*p* < 0.001) (Figure 2(a)), and the corresponding median OS was not reached and 15.9 months, respectively (*p* < 0.001) (Figure 2(b)). After PSM, the median PFS was 15.4 months in the TACE-MTAs-ICIs group and 4.8 months in the TACE group (*p* < 0.001) (Figure 3(a)), and the corresponding median OS was 27.2 and 15.9 months, respectively (*p* = 0.007) (Figure 3(b)).

After PSM, multivariate analysis indicated that the AFP level [hazard ratio (HR) = 2.96; 95% CI, 1.55–5.71; *p* = 0.001] and treatment method (HR = 0.31; 95% CI, 0.15–0.61; *p* = 0.001) were independent predictive factors for OS (Table 2). Multivariate analysis showed that the treatment method (HR = 0.23; 95% CI, 0.13–0.41; *p* < 0.001) was an independent predictive factor of PFS (Table 3).

**Table 2.** Predictive factor analysis for overall survival (after matching).

Variables	Univariate		Multivariate	
	HR (95% CI)	*p*	HR (95% CI)	*p*
Age (≥ 60 years)	0.77 (0.42–1.43)	0.411		
Sex (male)	1.06 (0.38–2.99)	0.907		
BCLC (B3)	4.81 (0.60–38.48)	0.139		
Child-Pugh class (B)	1.08 (0.48–2.43)	0.862		
ALBI (2)	1.16 (0.61–2.20)	0.657		
AFP (≥ 400 ng/mL)	2.58 (1.35–4.93)	0.004	2.96 (1.55–5.71)	0.001
Tumours number (≥ 3)	1.59 (0.84–3.03)	0.156		
Tumour diameter (≥ 7 cm)	0.93 (0.50–1.71)	0.809		
TACE-MTAs-ICIs	0.35 (0.18–0.70)	0.003	0.31 (0.15–0.61)	0.001

Abbreviations: BCLC, Barcelona Clinic Liver Cancer; ALBI, albumin-bilirubin; AFP, α‐fetoprotein.

**Table 3.** Predictive factor analysis for progression-free survival (after matching).

Variables	Univariate		Multivariate	
	HR (95% CI)	*p*	HR (95% CI)	*p*
Age (≥ 60 years)	0.86 (0.52–1.43)	0.579		
Sex (male)	0.82 (0.35–1.92)	0.652		
BCLC (B3)	1.23 (0.17–9.03)	0.836		
Child-Pugh class (B)	1.16 (0.58–2.28)	0.092	1.23 (0.60–2.54)	0.118
ALBI (2)	1.02 (0.60–1.74)	0.945		
AFP (≥ 400 ng/mL)	1.00 (0.61–1.65)	0.998		
Tumours number (≥ 3)	1.36 (0.82–2.27)	0.234		
Tumour diameter (≥ 7 cm)	0.78 (0.47–1.29)	0.780		
TACE-MTAs-ICIs	0.30 (0.18–0.51)	< 0.001	0.23 (0.13-0.41)	< 0.001

Abbreviations: BCLC, Barcelona Clinic Liver Cancer; ALBI, albumin-bilirubin; AFP, α‐fetoprotein.

### Tumour response

The tumour responses in the two groups after PSM are shown in Table 4. The ORR was significantly higher in the TACE-MTAs-ICIs group than in the TACE group (65.1% vs. 37.2%, *p* = 0.010). In addition, DCR was similar between the TACE-MTAs-ICI group and the TACE group (76.7% vs. 69.8%, *p* = 0.465).

**Table 4.** Tumour response based on the modified response evaluation criteria in solid tumours.

	Unmatched group			Matched group		
	TACE-MTAs-ICIs (*n* = 47)	TACE (*n* = 83)	*p* value	TACE-MTAs-ICIs (*n* = 43)	TACE (*n* = 43)	*p* value
CR	2 (4.3%)	1 (1.2%)	0.296	2 (4.7%)	1 (2.3%)	1.000
PR	28 (59.6%)	34 (41.0%)	0.041	26 (60.5%)	15 (34.9%)	0.018
SD	5 (10.6%)	21 (25.3%)	0.045	5 (11.6%)	14 (32.6%)	0.019
PD	12 (25.5%)	27 (32.5%)	0.403	10 (23.3%)	13 (30.2%)	0.465
ORR	30 (63.8%)	35 (42.2%)	0.018	28 (65.1%)	16 (37.2%)	0.010
DCR	35 (74.5%)	56 (67.5%)	0.403	33 (76.7%)	30 (69.8%)	0.465

Abbreviations: CR, complete response; PR, partial response; SD, stable response; ORR, objective response rate; PD, progressive disease; DCR, disease control rate; CI, confidence interval.

### Subgroup analysis

**Figure 4.**
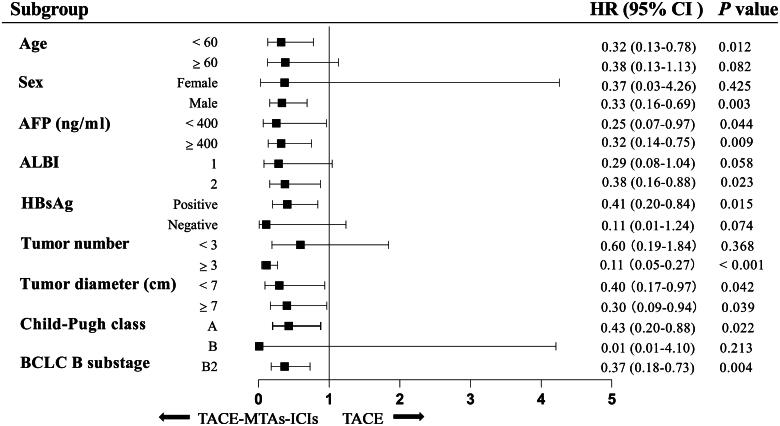
Forest plot for overall survival of the two groups of patients. Abbreviations: AFP, α-fetoprotein; ALBI, albumin-bilirubin; BCLC, Barcelona Clinic Liver Cancer; CI, confidence interval; HR, hazard ratio.

**Figure 5.**
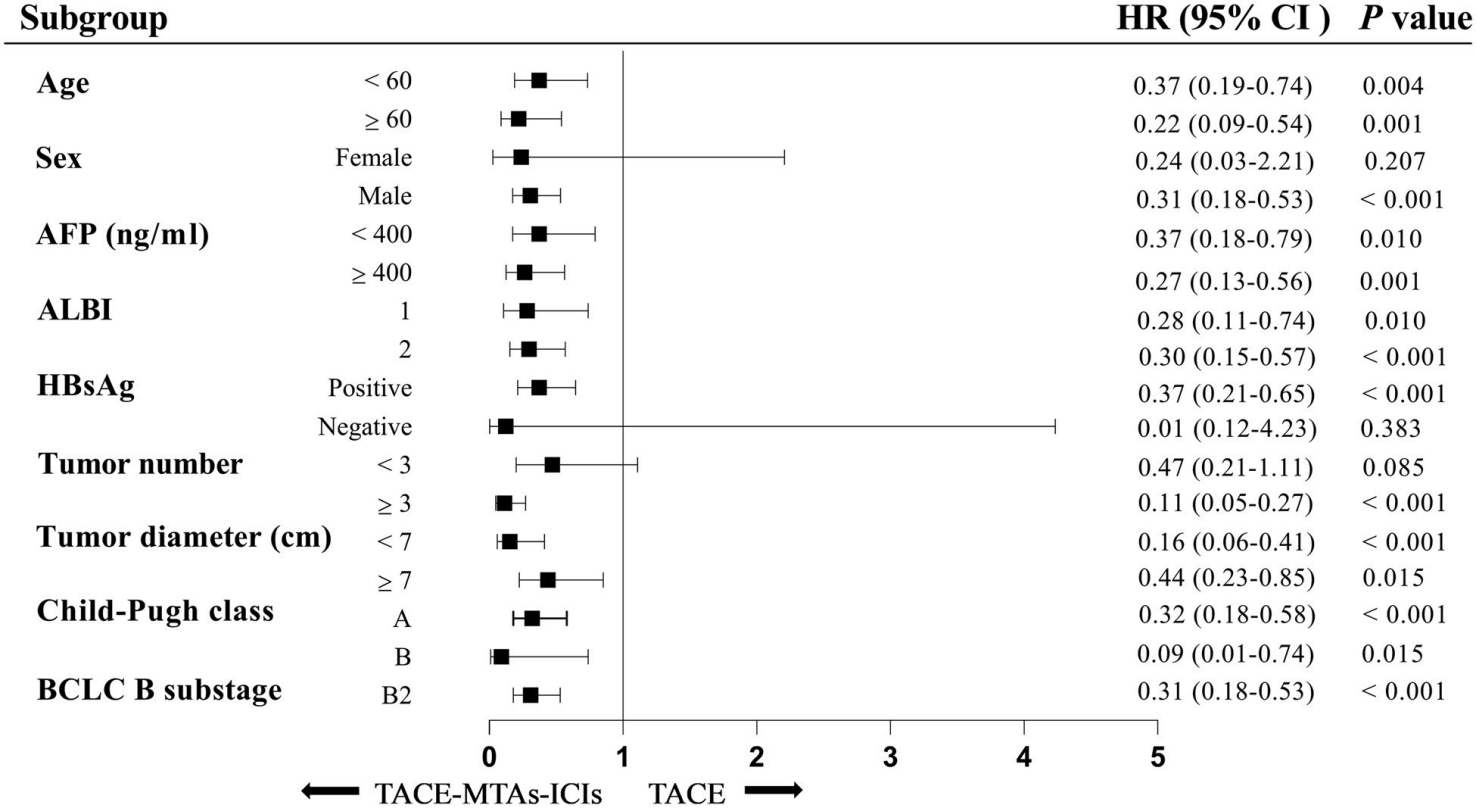
Forest plot for progression-free survival of the two groups of patients. Abbreviations: AFP, α-fetoprotein; ALBI, albumin-bilirubin; BCLC, Barcelona Clinic Liver Cancer; CI, confidence interval; HR, hazard ratio.

After PSM, the subgroup analyses of the factors associated with OS and PFS are shown in Figure 4 and Figure 5 as forest plot. The results indicated that TACE-MTAs-ICIs treatment could confer superior survival and PFS benefits for most subgroups.

### AEs (grades 3 and 4)

Treatment‐related AEs are shown in Table 5. The incidence rates of grade 3 and 4 AEs were higher in the TACE-MTAs-ICIs group than in the TACE group (83.7% vs. 51.2%, *p* = 0.001). These AEs resolved or were eliminated after conservative treatment. No treatment‐related deaths occurred. In addition, dose reduction and treatment interruption of MTAs were observed in 27.9% (12 of 43) of patients in the TACE-MTAs-ICIs group. Treatment interruption was performed in one patient in the TACE-MTA-ICI group because of hypothyroidism. After receiving symptomatic treatment, the patient resumed the previous treatment.

**Table 5.** Treatment-related adverse events (grade 3 or 4) (after matching).

Adverse events	TACE-MTAs-ICIs (*n* = 43)	TACE (*n* = 43)	*p* value
All	36 (83.7%)	22 (51.2%)	0.001
Hypertension	6 (13.2%)	0	0.026
Elevated AST	12 (27.9%)	9 (20.9%)	0.451
Elevated ALT	13 (30.2%)	9 (20.9%)	0.323
Hand-foot-skin reactions	1 (2.3%)	0	>0.999
Abdominal pain	2 (4.7%)	2 (4.7%)	>0.999
Fever	1 (2.3%)	2 (4.7%)	>0.999
Hypothyroidism	1 (2.3%)	0	>0.999

Abbreviations: AST, aspartate aminotransferase; ALT, alanine aminotransferase;

## Discussion

TACE is recommended as the best treatment for intermediate HCC in many countries [4]. However, HCC with BCLC B-stage is a highly heterogeneous, posing unique challenges for therapeutic modalities, and TACE might not be the most effective treatment for patients who are beyond the up-to-seven criteria [7, 9]. HCC patients who exceed the up-to-seven criteria are less likely to respond to TACE alone due to its high tumour burden [20]. In this study, compared to TACE alone, the triple treatment modality improved the tumour response rate (ORR: 65.1%) and clinical benefit (median OS: 27.2 months; median PFS: 15.4 months) in HCC patients who were beyond the up-to-seven criteria. Survival increased by a median of 11.3 months with the triple treatment modality.

The initial and optimal responses after TACE predict survival well. Tumours larger than 5 cm and ≥ 4 tumours are less likely to achieve a complete response to TACE [21]. Attaining an effective response at the initial TACE remains the strongest indicator of a favourable outcome in HCC, even after repeat sessions. Notably, repeated TACE procedures could impact liver function. Therefore, it is important to consider early initiation of systemic therapy to prevent any compromise in liver function, especially for HCC patients beyond the up-to-seven criteria, which could enhance overall treatment outcomes [5]. The TACTICS trial showed the benefit of sorafenib followed by TACE as a treatment option to improve the clinical outcome of patients with HCC compared with TACE alone (median PFS: 25.2 vs. 13.5 months, *p* = 0.006; OS at 1 year: 96.2% vs. 77.2%) [22]. Similarly, compared to monotherapy with lenvatinib, the combination of TACE and lenvatinib improved the median OS (17.8 vs. 11.5 months, *p* < 0.001) and median PFS (10.6 vs. 6.4 months, *p* < 0.001), as observed in the LAUNCH study [23]. Although phase III trials of immunotherapy with nivolumab or pembrolizumab did not meet the primary endpoint of OS, a clinical benefit was still observed [24, 25]. Combination therapies based on immunotherapy have been shown to improve survival rates and provide durable responses in HCC patients [26]. In particular, what we should learn from these studies is that the idea of treatment stage migration may be worth pursuing [4], and the early combination of TACE with MTAs and ICIs could result in extraordinary clinical benefits for intermediate-stage patients with a high tumour burden in the dawn of a promising new era for HCC treatment. Notably, TACE can also exacerbate microenvironmental hypoxia in tumour cells and trigger the upregulation of hypoxia-related factors, including VEGF and fibroblast growth factor [12, 13]. In addition, TACE can lead to locoregional tumour necrosis and release tumour antigens, which stimulates tumour-specific immune responses and neutralizes immune evasion [27]. MTAs may counteract hypoxia-induced angiogenesis after TACE and adjust the tumour immune microenvironment, boosting the immune response to PD-1 inhibitors in HCC [28].

Based on the potential mechanisms above, as in the protocol in our study, following the TACE procedure, combined MTAs and ICIs may cause a maximal surge of synergistic antitumor activity. Compared with TACE alone, triple therapy led to a higher tumour response rate (ORR: 65.1% vs. 37.2%, *p =* 0.010), a longer median PFS (15.4 vs. 4.8 months, *p* < 0.001), and a better median OS (27.2 vs. 15.9 months, *p* = 0.007) in HCC patients who were beyond the up-to-seven criteria. Not all intermediate-stage HCC patients benefit from TACE or combined therapy because it is a heterogeneous disease not only in terms of tumour burden but also in terms of liver function. In addition, it is important to preserve liver function and achieve a high objective response to prolong overall survival [5]. One study found that being beyond the up-to-seven criteria was associated with a decrease in the Child-Pugh grade after TACE [29]. In this study, the majority of the patients’ liver function was Child-Pugh A, which may be the maximum adaptation of the competitive risk of liver function damage caused by combined therapy or TACE. The combination of TACE with MTAs plus ICIs seems to be clinically feasible and safe [30–32]. Achieving a high ORR and preserving liver function are equally critical to prolong overall survival with HCC, especially on the background of liver cirrhosis [5]. In our study, the triple therapy group had a higher incidence of all treatment‐related AEs (grade ≥ 3 or 4). Overall, they were controllable and manageable. All approved molecularly targeted agents and immune checkpoint inhibitors are indicated only for patients with Child-Pugh A liver function [33]. Thus, a higher proportion of Child-Pugh A patients is associated with greater safety benefits from triple therapy.

This study had several natural limitations. First, the MTAs or ICIs used in the combined treatment group were not uniform. However, in general, the targets of ICIs or MTAs partially overlap. Therefore, it did not stifle the superiority of the triple modality. Second, nearly 90% of aetiologies were hepatitis B. The copies of the virus were monitored, and no reversal was found. Comprehensive monitoring in subsequent studies is required to further confirm the safety of the triple treatment protocol. Although a PSM analysis was used in this study, the potential selection bias could not be avoided entirely. Survival may be competitive, but the balance between liver function damage and patient tumour response benefit is not homogeneous in different cultural settings.

In conclusion, for uHCC patients beyond the up-to-seven criteria, TACE-MTAs-ICIs provided superior ORR and OS. Early combined TACE and systemic treatment should shift for patients who are beyond these criteria.

## Supplementary Material

Supplemental Material

## Data Availability

The data that support the findings of this study are available from the corresponding author [Q.Q. Z.], upon reasonable request.

## Abbreviations

Aes: adverse events
AFP: α-fetoprotein
ALBI: albumin-bilirubin
BCLC: Barcelona Clinic Liver Cancer
CI: confidence interval
CR: complete response
DCR: disease control rate
ECOG: Eastern Cooperative Oncology Group
HCC: hepatocellular carcinoma
HRs: hazard ratios
ICIs: immune checkpoint inhibitors
mRECIST: modified response evaluation criteria in solid tumours
MTAs: molecular-targeted agents
ORR: objective response rate
OS: overall survival
PFS: progression-free survival
PR: partial response
PSM: propensity score matching
SD: stable disease
TACE: transarterial chemoembolization
VEGF: vascular endothelial growth factor.
